# Glucagon resistance and metabolic-associated steatotic liver disease: a review of the evidence

**DOI:** 10.1530/JOE-23-0365

**Published:** 2024-04-27

**Authors:** Emma Rose McGlone, Stephen R Bloom, Tricia M-M Tan

**Affiliations:** 1 Department of Surgery and Cancer, Imperial College London, London, UK; 2 Department of Metabolism, Digestion and Reproduction, Imperial College London, London, UK

**Keywords:** glucagon, glucagon resistance, glucagon-like peptide 1, metabolic dysfunction-associated steatotic liver disease (MASLD), non-alcoholic fatty liver disease (NAFLD)

## Abstract

Metabolic-associated steatotic liver disease (MASLD) is closely associated with obesity. MASLD affects over 1 billion adults globally but there are few treatment options available. Glucagon is a key metabolic regulator, and its actions include the reduction of liver fat through direct and indirect means. Chronic glucagon signalling deficiency is associated with hyperaminoacidaemia, hyperglucagonaemia and increased circulating levels of glucagon-like peptide 1 (GLP-1) and fibroblast growth factor 21 (FGF-21). Reduction in glucagon activity decreases hepatic amino acid and triglyceride catabolism; metabolic effects include improved glucose tolerance, increased plasma cholesterol and increased liver fat. Conversely, glucagon infusion in healthy volunteers leads to increased hepatic glucose output, decreased levels of plasma amino acids and increased urea production, decreased plasma cholesterol and increased energy expenditure. Patients with MASLD share many hormonal and metabolic characteristics with models of glucagon signalling deficiency, suggesting that they could be resistant to glucagon. Although there are few studies of the effects of glucagon infusion in patients with obesity and/or MASLD, there is some evidence that the expected effect of glucagon on amino acid catabolism may be attenuated. Taken together, this evidence supports the notion that glucagon resistance exists in patients with MASLD and may contribute to the pathogenesis of MASLD. Further studies are warranted to investigate the direct effects of glucagon on metabolism in patients with MASLD.

## Introduction

Glucagon is a peptide hormone secreted by alpha cells in the pancreas which acts on several organs including the liver, kidney, heart and brain (Marroqui *et al.* 2014, Wewer Albrechtsen *et al.* 2023). The precursor protein transcribed from the proglucagon gene is processed by the enzyme prohormone convertase 1 and 2 to give glucagon and related peptides including glucagon-like peptide 1 (GLP-1) and oxyntomodulin (Müller *et al.* 2017). Glucagon regulates glucose, protein and lipid metabolism, but its actions can be obscured by those of other hormones, notably insulin and GLP-1.

Metabolic-associated steatotic liver disease (MASLD, formerly known as non-alcoholic fatty liver disease (NAFLD)) affects one-third of the global population and can lead to steatohepatitis, cirrhosis, liver failure and hepatocellular cancer (Younossi *et al.* 2016). It has been proposed that patients with MASLD are resistant to glucagon (Richter *et al.* 2022, Wewer Albrechtsen *et al.* 2023). Since one of the actions of glucagon is to decrease liver fat, resistance to glucagon could contribute to the pathological accumulation of liver fat seen in MASLD. To investigate resistance to glucagon, we must first review the metabolic actions of glucagon in health. Here the phenotype of glucagon-signalling deficiency will be explored, focusing on glucagon receptor knockout (GCGR^−/−^) mice, followed by the metabolic effects of glucagon infusion, with the aim of determining what metabolic phenotype would be expected in glucagon resistance as baseline and when stimulated with glucagon. We will review the evidence that patients with MASLD exhibit these features.

## Glucagon signalling deficiency

Chronic absence of glucagon signalling occurs in Mahvash disease, caused by a homozygous missense mutation in the GCGR, which leads to a 96% reduction in glucagon binding (Zhou *et al.* 2009). This very rare condition (4 per million) is associated with marked hyperglucagonaemia (100–1000 fold) and alpha-cell hyperplasia, with the formation of glucagonomas and ultimately pancreatic neuroendocrine tumours (PNETs) (Zhou *et al.* 2009, Yu 2018). Apart from requiring monitoring and treatment for PNETs, patients are otherwise clinically well. They may have moderate hepatosteatosis (Robbins *et al.* 2023). Notably, hypoglycaemia is not clinically significant.

**Figure 1:**
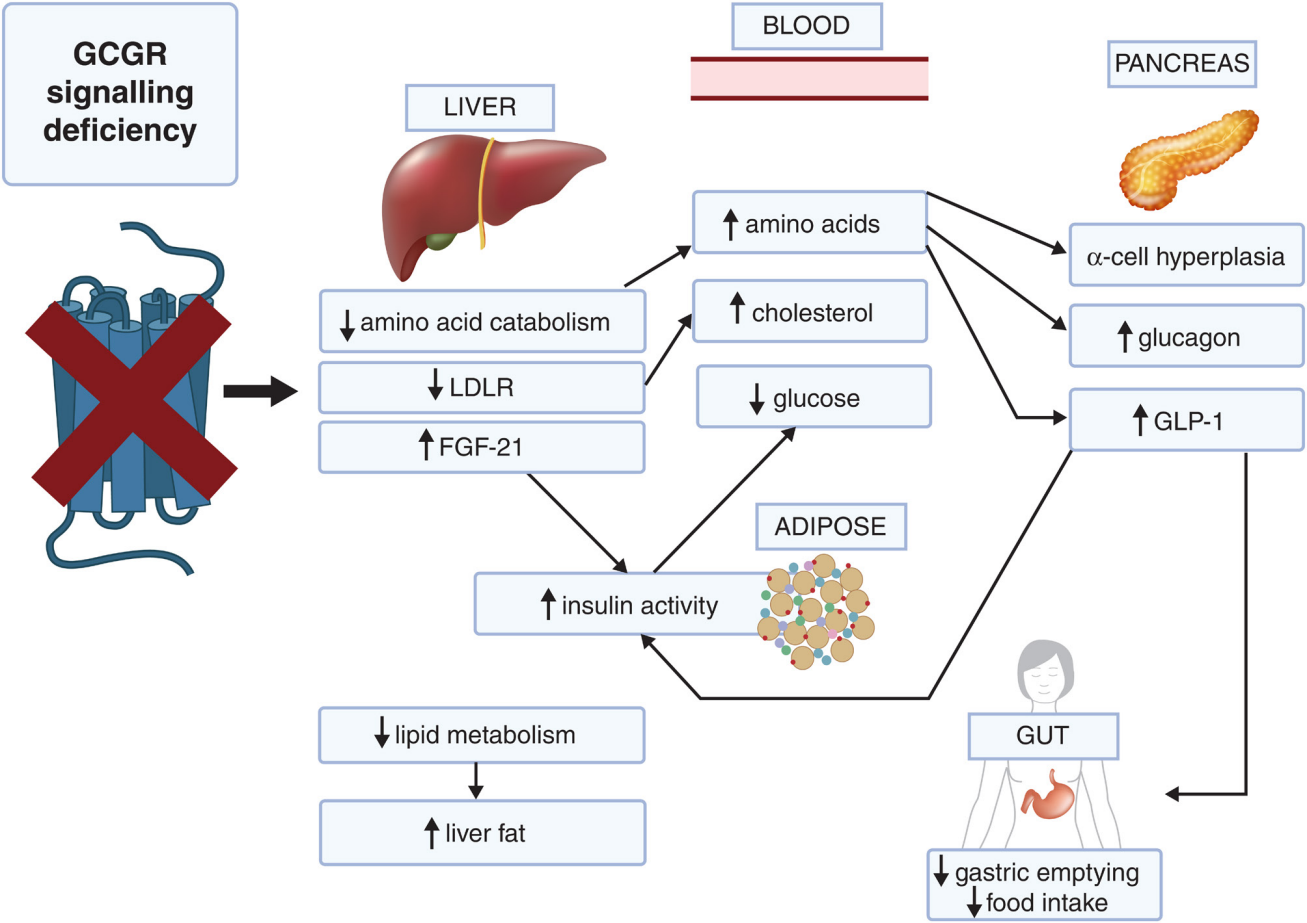
Major effects of glucagon signalling deficiency. Glucagon receptor (GCCR) blockade decreases hepatic expression of amino acid catabolism and uptake enzymes, leading to hyperaminoacidaemia. It also decreases expression of low-density lipoprotein receptor (LDLR), which reduces hepatic cholesterol uptake. Amino acids stimulate alpha cells in the pancreas, leading to their hyperplasia and increased transcription of the preproglucagon gene, which in turn leads to increases in circulating glucagon and glucagon-like peptide 1 (GLP-1). There are increased levels of hepatic and plasma fibroblast growth factor 21 (FGF-21). Reduced hepatic glucagon receptor signalling decreases expression of lipid metabolism genes, leading to an increase in liver fat, and decreases in hepatic glucose production. Glucose tolerance is also improved indirectly, via increases in GLP-1 and FGF-21 which improve insulin sensitivity and availability. GLP-1 also acts on the brain and stomach to decrease food intake and decrease gastric emptying. Created with BioRender.com.

Individuals with Mahvash disease have a phenotype very similar to that observed in mice lacking the glucagon receptor (GCGR^−/−^). Such mice have dramatically raised glucagon levels (100-fold) accompanied by extreme hypertrophy of the pancreas and hyperplasia of islets (Parker *et al.* 2002, Gelling *et al.* 2003, Conarello *et al.* 2007, Yuan *et al.* 2023). Pharmacological inhibition of glucagon signalling (e.g. GCGR antagonists or GCGR antisense oligonucleotides) also increases circulating glucagon levels and causes alpha-cell hyperplasia (Sloop *et al.* 2004, Winzell *et al.* 2007). Chronic glucagon signalling deficiency is also associated with elevated plasma GLP-1 (10- to 25-fold), which is likely due to alpha-cell hyperplasia and increased transcription of preproglucagon (Parker *et al.* 2002, Gelling *et al.* 2003, Sloop *et al.* 2004, Yuan *et al.* 2023). GCGR^−^^/−^ mice have elevated plasma fibroblast growth factor 21 (FGF-21) (Omar *et al.* 2013), which cannot be due to hyperglucagonaemia because glucagon normally increases plasma and hepatic expression of FGF-21 via a direct effect on the glucagon receptor (Habegger *et al.* 2013, Cyphert *et al.* 2014). The underlying mechanism for the elevated FGF-21 in GCGR^−/−^ mice has not been established, but one possibility is that it occurs secondary to elevated GLP-1 levels, since GLP-1R agonists increase plasma and hepatic FGF-21 in a pathway dependent on central neuronal GLP-1R and hepatic peroxisome proliferator-activated receptor (Le *et al.* 2023). Other hormonal changes exhibited by GCGR^−/−^ mice are a reduction in leptin, presumably due to lower fat mass, increase in ghrelin (two- to three-fold) and heightened sensitivity to epinephrine (Gelling *et al.* 2003, Mani *et al.* 2017). Some of the metabolic changes described below are not directly attributable to a reduction in glucagon signalling but occur indirectly, via changes in the activity of other hormones (Fig. 1).

### Amino acid catabolism

Hepatocyte-specific GCGR knockout mice exhibit similar phenotype to the whole body knockout (Longuet *et al.* 2013), including hyperglucagonaemia and alpha-cell hyperplasia. This finding led researchers to suspect that the alpha-cell hyperplasia seen in GCGR^−/−^ mice was due to a factor secreted by the liver and carried in the circulation. Indeed, alpha cell proliferation is observed in wildtype mouse islets cultured in serum from GCGR^−/−^ mice (Dean *et al.* 2017).

Transcriptomic and metabolomic analyses performed to reveal the responsible circulating factor(s) demonstrated firstly that GCGR^−/−^ mice have decreased expression of hepatic amino acid catabolism enzymes (Yang *et al.* 2011, Solloway *et al.* 2015, Dean *et al.* 2017, Winther-Sørensen *et al.* 2020), as do GCGR^−/−^ zebrafish which have a similar phenotype to GCGR^−/−^ mice including alpha-cell hyperplasia (Kang *et al.* 2020). Secondly, GCGR^−/−^ mice have increased circulating amino acids by 2–10-fold (Yang *et al.* 2011, Solloway *et al.* 2015, Galsgaard *et al.* 2018).

Similarly, pharmacological blockage of the GCGR in mice is associated with elevated plasma amino acids and decreased transcription of amino acid uptake and ureagenesis enzymes (Galsgaard *et al.* 2019, Galsgaard *et al.* 2020, Elmelund *et al.* 2022). In man, chronic glucagon deficiency secondary to total pancreatectomy (Boden *et al.* 1980) and acute glucagon deficiency due to somatostatin infusion (Boden *et al.* 1984) are also associated with elevated levels of plasma amino acids. Although the species of amino acid elevated varies depending on experimental technique, alanine, arginine and glycine are consistently elevated. In the very rare context of reduced glucagon signalling secondary to Mahvash disease, plasma glutamine, citrulline, orthinine and arginine have been noted to be elevated (Robbins *et al.* 2023).

Amino acids stimulate pancreatic secretion of glucagon *in vitro* and *in vivo*, in particular alanine, arginine, cysteine and proline (Galsgaard *et al.* 2018, Galsgaard *et al.* 2020). In both zebrafish and mice, the amino acid-stimulated proliferation of alpha cells is mediated by the mammalian target of rapamycin (mTor) (Solloway *et al.* 2015, Dean *et al.* 2017).

Taken together, this evidence suggests that GCGR signalling deficiency leads to a downregulation of glucagon-stimulated hepatic amino acid uptake and catabolism, leading to hyperaminoacidaemia, which in turn stimulates the pancreas and causes profound alpha-cell hyperplasia and increased transcription of preproglucagon, with resulting hyperglucagonaemia and increased GLP-1.

### Glucose tolerance

Mice lacking the glucagon receptor have lowered fasting blood glucose compared to wild-type counterparts (Parker *et al.* 2002, Gelling *et al.* 2003). When challenged with oral or intra-peritoneal glucose, both lean and obese GCGR^−/−^ mice have improved glucose tolerance (Parker *et al.* 2002, Gelling *et al.* 2003, Conarello *et al.* 2007). Pharmacological inhibition of glucagon receptor signalling in rodent models is also consistently associated with improved glucose tolerance (Lasher *et al.* 2022). Early clinical trials of glucagon receptor antagonists also demonstrated reduced fasting and post-prandial glucose levels in healthy volunteers and patients with type 2 diabetes (Petersen & Sullivan 2001, Guan *et al.* 2015, Kelly *et al.* 2015).

Lean GCGR^−/−^ mice have lower fasting and fed insulin levels than wild-type littermates (Conarello *et al.* 2007), although this is not statistically significant in all studies (Parker *et al.* 2002, Gelling *et al.* 2003, Yuan *et al.* 2023). Although islets from mice treated with glucagon receptor antagonists have increased insulin content and glucose-stimulated insulin secretion, with mice mounting a greater insulin response to a glucose tolerance test (Sloop *et al.* 2004, Winzell *et al.* 2007), GCGR^−/−^ mice behave comparably to controls during insulin tolerance tests (Yuan *et al.* 2023). These findings suggest that decreased glucagon signalling does not directly affect insulin sensitivity but augments insulin availability. This is likely an indirect effect of elevated GLP-1, which powerfully augments glucose-stimulated insulin secretion (MacDonald & Rorsman 2023). Additionally, GCGR^−/−^ mice exhibit slowed gastric emptying, also potentially due to elevated GLP-1 (Conarello *et al.* 2007).

Injection of streptozotocin (STZ), a beta cell toxin, models type 1 diabetes mellitus in mice, causing hyperglycaemia, polyuria, polydipsia and cachexia. GCGR^−/−^ mice, however, remain euglycaemic and healthy following STZ injection (Conarello *et al.* 2007, Lee *et al.* 2011). Initially this was thought to be due to a resistance to the beta cell toxicity of STZ in GCGR^−/−^ mice because insulin levels were not suppressed following STZ injection (Conarello *et al.* 2007). Further investigation, however, demonstrated that resistance to hyperglycaemia persists in GCGR^−/−^ mice even after a double dose of STZ ensures dramatic reduction in circulating insulin (Lee *et al.* 2011). In situations, however, where there is no beta cell function whatsoever (diphtheria toxin induced beta cell loss or insulin receptor knockout), lack of GCGR does not prevent diabetes or improve survival (Damond *et al.* 2016, Neumann *et al.* 2016). These findings indicate that the glycaemic effect of glucagon receptor absence is highly dependent on beta cell status.

In contrast to mice with congenital GCGR^−/−^, mice with an inducible GCGR^−/−^ knockdown treated with STZ have only partial protection from hyperglycaemia (Rivero-Gutierrez *et al.* 2018). The resistance to STZ-induced diabetes is therefore likely due to chronic compensatory mechanisms in GCGR^−/−^ mice, mediated by elevation of GLP-1, which increases glucose-stimulated insulin secretion, and FGF-21, which promotes beta cell regeneration and increases peripheral glucose uptake (Omar *et al.* 2013, Cui *et al.* 2023).

### Body composition

On a standard diet, mice lacking the glucagon receptor resemble wild-type controls. They have similar whole body and liver weights, food intake, resting energy expenditure and respiratory quotients (Gelling *et al.* 2003, Yuan *et al.* 2023). They have slightly higher lean mass relative to fat mass (Gelling *et al.* 2003, Conarello *et al.* 2007, Yuan *et al.* 2023). When challenged with a high fat diet, however, they are resistant to weight gain and associated hyperinsulinaemia (Conarello *et al.* 2007). They exhibit a much lower increase in white and brown adipose mass, with preservation of lean mass (Conarello *et al.* 2007, Longuet *et al.* 2008). This may be explained by a lower food intake than their wild-type counterparts (Conarello *et al.* 2007), which is counterintuitive because glucagon receptor agonism decreases food intake in rodents (Inokuchi *et al.* 1984, Geary *et al.* 1993). A decrease in food intake is therefore likely to occur secondary to elevated GLP-1 levels (Nakatani *et al.* 2017).

### Lipid metabolism

After a short period of fasting, lean GCGR^−/−^ mice have similar levels of plasma triglycerides, free fatty acids and cholesterol to wild-type mice (Parker *et al.* 2002, Gelling *et al.* 2003, Conarello *et al.* 2007, Longuet *et al.* 2008). After a prolonged fast, however, knockout mice have inappropriately high fasting triglycerides and free fatty acids. GCGR^−/−^ mice fail to upregulate hepatic fatty acid oxidation pathways in response to endogenous (secondary to fasting) or exogenously administered glucagon (Longuet *et al.* 2008). Hepatic transcriptomics reveals downregulation of genes related to fatty acid beta-oxidation and increases in genes for fatty acid synthesis in GCGR^−/−^ mammals (Yang *et al.* 2011, Dean *et al.* 2017, Kang *et al.* 2020). These changes results in propensity to hepatosteatosis in most reports (Longuet *et al.* 2008, Kang *et al.* 2020), but not all (Conarello *et al.* 2007). This discrepancy could be because ectopic liver fat is closely related to overall body weight and so the resistance to overall weight gain in GCGR^−/−^ mice is a major confounder.

Acute treatment with glucagon receptor antagonists is however consistently associated with an increase in liver transaminases and liver fat, which is dose-dependent and reversible on drug cessation (Guan *et al.* 2015, Kelly *et al.* 2015, Guzman *et al.* 2017, Kostic *et al.* 2018). This is now accepted to be an ‘on-target’ effect attributable to glucagon’s role in decreasing liver fat accumulation and has resulted in these medications being withdrawn from further clinical development (Novikoff & Muller 2023).

In mice with diet induced obesity, inducible hepatocyte GCGR^−/−^ knockdown leads to elevated plasma cholesterol associated with a reduction in hepatic low-density lipoprotein receptor (LDLR) expression (Spolitu *et al.* 2019). The underlying mechanism may be an increase in hepatic and plasma levels of proprotein convertase subtilisin/kexin type 9 (PCSK9), the enzyme which degrades LDLR (Spolitu *et al.* 2019). Patients treated with glucagon receptor antagonists also have increased plasma cholesterol; as well as its direct hepatic effects, glucagon antagonism may also increase intestinal cholesterol absorption (Guan *et al.* 2015).

## Acute glucagon infusion

Patients with glucagonoma (glucagon-secreting tumours of the pancreas) exhibit profound hypoaminoacidaemia, mild hyperglycaemia and weight loss (Bloom & Polak 1987, Almdal *et al.* 1990, Klein *et al.* 1992, Bernstein *et al.* 2001). They commonly suffer from necrolytic migratory erythema, which is a skin rash characterised by superficial epidermal necrosis (Pedersen *et al.* 1976). This is likely to be caused by extremely low circulating amino acids, as it resolves when mixed amino acids are infused (Holst *et al.* 2017). Glucagonoma syndrome is rare and case studies give conflicting results regarding its effects on metabolism. For example, basal rates of glucose production may be increased (Klein *et al.* 1992), or unchanged (Boden *et al.* 1978). An isotope study in a patient with glucagonoma demonstrated minimal abnormalities in net protein breakdown when compared to healthy volunteers but a possible increase in amino acid oxidation. During amino acid infusion, amino acid clearance and ureagenesis were increased (Almdal *et al.* 1990). This patient had increased whole-body lipolysis rates as measured by rate of glycerol appearance, which was accompanied by an increase in the rate of re-esterification to triglyceride (Almdal *et al.* 1990). A study of a second patient indicated enhanced rates of ketogenesis but no change in lipolysis (Boden *et al.* 1978). Differences in the clinical picture may occur because patients with glucagonomas are also chronically malnourished (negative energy balance causes a decrease in rates of protein synthesis and breakdown, which could counter a catabolic effect of glucagon); they also have markedly raised levels of circulating insulin and free fatty acids (Klein *et al.* 1992, Bernstein *et al.* 2001); and they have been exposed to glucagon excess for variable durations of time.

Glucagon infusions in healthy volunteers provide a means of examining acute effects of glucagon in different nutritional contexts and disease states. They also allow for fixing of blood levels of other metabolically active substrates. The effects of glucagon infusion on healthy volunteers can be categorised into those on glucose, protein and lipid metabolism, and overall energy balance (Table 1).

**Table 1:** Key metabolic effects of acute glucagon infusion.

	Healthy volunteers	Subjects with obesity/MASLD	Differences between cohorts
Glucose	↑ plasma glucose, ↑ plasma insulin (Lockton & Poucher 2007) ↑ hepatic glucose production (HGP) (Chiasson *et al.* 1975)	↑ plasma glucose, ↑ plasma insulin (Vega *et al.* 2021) ↑ HGP (Suppli *et al.* 2020)	No difference in HGP incremental change observed between healthy volunteers vs patients with obesity (Suppli *et al.* 2020); or patients with MASLD vs weight-matched controls (Heebøll *et al.* 2022)
Protein	↓↓ plasma amino acids (Boden *et al.* 1984) ↑ urea production (Fabbri *et al.* 1993, Hamberg & Vilstrup 1994)	↓ (Vega *et al.* 2021) or slight ↑ plasma amino acids (Suppli *et al.* 2020) ↑urinary nitrogen excretion (Vega *et al.* 2021)	Attenuated reduction in amino acids in patients with obesity (+/-MASLD) vs healthy volunteers (Suppli *et al.* 2020)
Lipid	↑ plasma-free fatty acids (Arafat *et al.* 2013, Liljenquist *et al.* 1974) ↓plasma cholesterol (Xiao *et al.* 2011)	↓ plasma VLDL–triglyceride (Heebøll *et al.* 2022)	Attenuated reduction in plasma VLDL–triglyceride in patients with MASLD vs weight-matched controls (Heebøll *et al.* 2022)
Energy balance	↑ energy expenditure (Salem *et al.* 2016) ⇔ energy intake (Frampton *et al.* 2022)	↑ energy expenditure (Cegla *et al.* 2014) ⇔ energy intake (Cegla *et al.* 2014)	No studies

Key effects of acute glucagon infusion in healthy volunteers, patients with obesity and/or metabolic-associated steatotic liver disease (MASLD), and evidence for differences between the two groups. Only select references included, for more please see main text.

HGP, hepatic glucose production; VLDL, very low-density lipoprotein.

### Effects on glucose and insulin

In fasted healthy volunteers, glucagon infusion leads to an increase in plasma glucose peaking at 20–30 min, accompanied by an increase in plasma insulin (Chiasson *et al.* 1975, Lockton & Poucher 2007, Salem *et al.* 2016). A similar picture is seen when glucagon is injected alongside an amino acid infusion which raises plasma amino acid concentration to high physiological levels (Kjeldsen *et al.* 2023*b*), as well as post-prandially (Bagger *et al.* 2015).

The observed rise in insulin is not solely secondary to the increase in plasma glucose. Glucagon is known to be insulinotropic, particularly in the mildly hyperglycaemic or fed state (Capozzi *et al.* 2019*a*, *b*). This effect is likely to be mediated through both GCGR and GLP-1 receptors on pancreatic beta cells, and it occurs even at low levels of glucagon (which do not increase hepatic glucose production) (Caruso *et al.* 2023).

Where somatostatin is infused alongside a glucagon infusion, to block endogenous pancreatic hormone secretion, the glucagon-stimulated rise in insulin is blocked, potentially resulting in a more pronounced blood glucose rise (Nair *et al.* 1987). Similarly, in patients with pancreatic deficiency secondary to type 1 diabetes, the effect of intravenous glucagon on AUC of glucose over 240 min is greater than in healthy volunteers (Arafat *et al.* 2013). Conversely, when insulin is co-infused to produce hyperinsulinaemia, hepatic glucose output is suppressed, even in the presence of a glucagon infusion; however, the addition of amino acids to the glucagon infusion can overcome the effect of hyperinsulinaemia and increase hepatic glucose output (Boden *et al.* 1990).

The rise in plasma glucose seen after glucagon infusion reflects increases in hepatic glucose production (Chiasson *et al.* 1975, Boden *et al.* 1984). Using arteriovenous difference experiments in dogs (in which the portal vein and the brachial artery are cannulated), hepatic glucose output can be directly measured (Cherrington 1999). In combination with a glucose tracer to measure hepatic glucose uptake, and measurements of gluconeogenic precursors and lactate, rates of glycogenolysis and gluconeogenesis can be derived. In dogs, where levels of insulin were clamped at basal, the increase in hepatic glucose production in response to hyperglucagonaemia was estimated to be entirely due to increases in glycogenolysis with no change in gluconeogenesis (Cherrington 1999). With the same experimental set-up, glucagon deficiency was associated with a decrease in glycogenolysis and no change in gluconeogenesis (Cherrington 1999). In fasted volunteers with unclamped insulin (Chiasson *et al.* 1975) and in fed individuals with euglycemic hyperinsulinaemia (Boden *et al.* 1990), however, gluconeogenesis increases in response to glucagon infusion, and this is due to a doubling of the rate of gluconeogenesis from alanine (Chiasson *et al.* 1975).

The rise in hepatic glucose production and glycogenolysis in response to a glucagon infusion peaks at around 15 min and then decreases (but remains by about 40% of its baseline value) by 2–3 h (Chiasson *et al.* 1975, Ramnanan *et al.* 2011).

### Effects on amino acid turnover and ureagenesis

In healthy volunteers infusion of glucagon decreases total plasma amino acids (Fitzpatrick *et al.* 1977, Boden *et al.* 1984, Boden *et al.* 1990, Suppli *et al.* 2020). This occurs even when glucose levels are clamped (Boden *et al.* 1984). It also persists in the absence of insulin (Nair *et al.* 1987); however, in insulin-deficient individuals, where there is no increase in insulin secretion following glucagon infusion, the reduction in branched chain amino acids (isoleucine, leucine and valine) is attenuated (Liljenquist *et al.* 1981). In dogs, where insulin was clamped at basal levels, glucagon infusion did not lead to any change in BCAAs (Kraft *et al.* 2017). The amino acids most significantly decreased by glucagon infusion vary between studies, but usually include alanine, arginine, threonine, proline and glycine (Fitzpatrick *et al.* 1977, Boden *et al.* 1984, Nair *et al.* 1987, Boden *et al.* 1990).

Hypoinsulinaemia causes an increase in whole body proteolysis as measured by leucine turnover (leucine is an essential amino acid so dilution of a tracer with unlabelled leucine is assumed to be a measure of endogenous whole body protein catabolism) (Nair *et al.* 1987). Infusion of glucagon in hypoinsulinaemic volunteers further increases proteolysis, as well as increasing leucine oxidation. In dogs, hyperglucagonaemia decreases protein synthesis and increases hepatic expression of phosphoenolpyruvate carboxykinase (PEPCK), catalysing gluconeogenesis from amino acids, and carbamoyl phosphate synthetase 1 (CPS1), catalysing urea synthesis (Flakoll *et al.* 1994, Kraft *et al.* 2017). Infusion of glucagon increases urea production rate in healthy volunteers (Boden *et al.* 1990, Fabbri *et al.* 1993, Hamberg & Vilstrup 1994) and dogs (Kraft *et al.* 2017) undergoing amino acid infusion. Urinary urea and nitrogen excretion are significantly lower during acute glucagon deficiency than excess (Boden *et al.* 1984).

Taken together, these studies indicate that glucagon promotes uptake and catabolism of circulating amino acids, including using them as fuel for gluconeogenesis, and increasing urea excretion. It is likely that the direct effect of glucagon infusion pertains to glucogenic amino acids, but is accompanied by a small decrease in branched chain amino acids secondary to the increase in insulin.

### Effects on hepatic lipid metabolism

Glucagon infusions decrease plasma cholesterol (Aubry *et al.* 1974, Xiao *et al.* 2011). This is likely to be mediated by an increase in low-density lipoprotein binding to its hepatic receptor, thereby increasing cholesterol uptake (Brown *et al.* 1989), and a reduction in amino acid incorporation into hepatic lipoproteins (Eaton 1973, Xiao *et al.* 2011).

Despite the robust findings that inhibiting glucagon activity directly increases hepatic lipid accumulation (see the ‘Lipid metabolism’ section), there is little data available of the effects of acute glucagon infusion on hepatic lipolysis. This is largely due to the difficulty of obtaining liver tissue in human subjects. In lactating cows treated with continuous glucagon infusion or vehicle, the former group experienced a concurrent increase in plasma insulin, and liver triacylglycerol fell over time post partum comparably in both groups (She *et al.* 1999). In another study where lactating cows were fed additional concentrate to induce fatty liver disease, subsequent glucagon infusion did not change insulin levels and in this context was associated with a significant reduction in liver triacylglycerol when compared to vehicle-treated cows (by 71% over 14 days) (Hippen *et al.* 1999).

### Effects on peripheral lipid metabolism

Lipolysis, the breakdown of triglyceride to glycerol and fatty acids, is strongly inhibited by insulin (Jaworski *et al.* 2007). Thus a glucagon-stimulated increase in insulin is associated with a decrease in plasma glycerol and non-esterified fatty acid (NEFA) concentrations (Liljenquist *et al.* 1974, Arafat *et al.* 2013, Heebøll *et al.* 2022). In the absence of insulin (patients with type 1 diabetes), however, glucagon administration causes an increase in plasma NEFA, glycerol and splanchnic ketone production (Liljenquist *et al.* 1974, Arafat *et al.* 2013); while suppression of glucagon by somatostatin leads to a decrease in free fatty acids and glycerol (Gerich *et al.* 1975). Similarly, where insulin is clamped at basal levels, hyperglucagonaemia is associated with increased rate of appearance of glycerol and NEFAs, whereas hypoglucagonaemia is associated with relative suppression (Carlson *et al.* 1993). It therefore seems likely that in healthy subjects, the antilipolytic effects of insulin overwhelm the lipolytic effects of glucagon (Liljenquist *et al.* 1974).

Although in rat adipocytes glucagon stimulates the release of glycerol (Arafat *et al.* 2013), indicating a direct effect on lipolysis, it is unlikely that the GCGR is expressed in murine or human white adipose tissue (Bomholt *et al.* 2022). When glucagon is infused into abdominal adipose wall tissue in healthy volunteers, alongside somatostatin and basal insulin, there is no change in local levels of glycerol (Gravholt *et al.* 2001). Glucagon’s stimulation of peripheral lipolysis could be mediated via increases in FGF-21 or GLP-1, both of which may be directly lipolytic in adipocytes (Villanueva-Peñacarrillo *et al.* 2001, Hotta *et al.* 2009). Further, glucagon stimulates secretion of catecholamines and cortisol, which could also increase lipolysis peripherally (Jones *et al.* 2012).

### Effects on energy expenditure and intake

Acute glucagon infusion (<3 h) leads to an increase in energy expenditure in normal weight (Salem *et al.* 2016, Chakravarthy *et al.* 2017) and overweight individuals (Tan *et al.* 2013, Cegla *et al.* 2014) as measured by indirect calorimetry. The mechanism through which this occurs does not appear to be via an increase in brown adipose tissue activation (Salem *et al.* 2016). Experiments with chronic administration of a glucagon analogue in mice indicate that the observed increase in energy expenditure is dependent on hypoaminoacidaemia, as it can be reversed by amino acid supplementation (Hope *et al.* 2022). It is worth noting, however, that an increase in energy expenditure was not reported following chronic administration of glucagon (72 h) in individuals with overweight/obesity (Whytock *et al.* 2021).

With respect to food intake, while one acute glucagon infusion study reported an increase (Izzi-Engbeaya *et al.* 2020), others have reported a decrease (Geary *et al.* 1992) or no change (Cegla *et al.* 2014). Recent meta-analysis of results demonstrated no significant effect (Frampton *et al.* 2022).

## Evidence for glucagon resistance in patients with MASLD

It has been proposed that patients with MASLD are resistant to glucagon activity (Richter *et al.* 2022, Wewer Albrechtsen *et al.* 2023). Since blocking the actions of glucagon increases liver fat (see above), it is feasible that glucagon resistance could contribute to the pathophysiology of MASLD. In this section, we will review evidence that patients with MASLD have a metabolic phenotype consistent with glucagon resistance, at baseline, and when stimulated with glucagon infusions.

**Figure 2:**
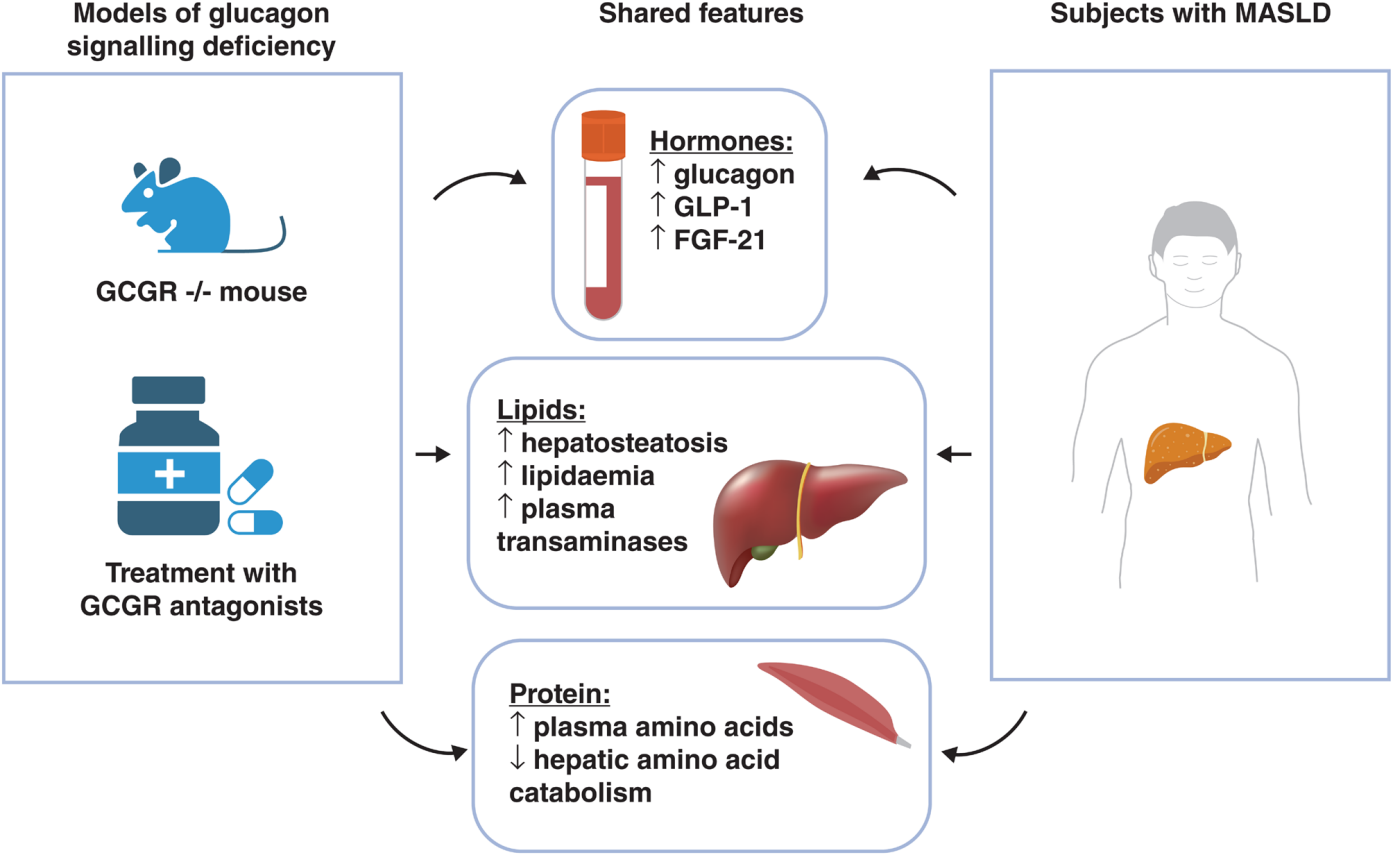
Shared features between models of glucagon signalling deficiency and patients with metabolic-associated steatotic liver disease. GCGR^−/−^, glucagon receptor knockdown; GLP-1, glucagon-like peptide 1; FGF-21, fibroblast growth factor 21; MASLD, metabolic-associated steatotic liver disease. Created with BioRender.com.

Patients with MASLD share several phenotypic features with pharmacological and genetic models of glucagon signalling deficiency (Fig. 2). As well as excessive liver fat, often associated with increased liver transaminases, they commonly suffer from lipidaemia and may have increased hepatic expression of PCSK9 (Rinella *et al.* 2023, Castellano-Castillo *et al.* 2024). They also have changes in circulating hormones and amino acids as described in this section.

### Hyperglucagonaemia and hyperaminoacidaemia

As noted earlier in this review, amino acids are a powerful stimulus to glucagon secretion from pancreatic alpha cells. Whereas carbohydrate consumption in healthy individuals leads to suppression of glucagon secretion, protein meals powerfully increase glucagon levels (Day *et al.* 1978). Post-prandially, a rise in both glucagon and insulin levels increases glucose flux with insulin-driven glucose uptake and glucagon-driven glucose production, resulting in euglycaemia (Ang *et al.* 2019). The liver–alpha cell axis describes a feedback loop, tightly regulated in health, between hepatocytes and alpha cells in the pancreas (Holst *et al.* 2017, Richter *et al.* 2022). Circulating amino acids stimulate pancreatic alpha cells to secrete glucagon, which increases hepatic amino acid uptake and catabolism, thereby reducing amino acid levels in the blood and reducing the stimulus for glucagon secretion. Conversely, high plasma levels of glucose inhibit glucagon secretion, which decreases hepatic glucose production, reduces hyperglycaemia and decreases the inhibition of glucagon secretion.

Where glucagon signalling is disrupted, the feedback system fails, resulting in hyperglucagonaemia and hyperaminoacidaemia (Holst *et al.* 2017, Richter *et al.* 2022). It has been observed in many studies that patients with MASLD do exhibit both hyperglucagonaemia and hyperaminoacidaemia (Junker *et al.* 2016, Wewer Albrechtsen *et al.* 2018*a*, Grandt *et al.* 2023). Fasting glucagon levels are around 1.5- to 2-fold higher than in healthy volunteers: 7.5–9 pmol/L vs 3.4–6 pmol/L, depending on the study (Junker *et al.* 2016, Pedersen *et al.* 2020, Grandt *et al.* 2023). One hypothesis is that excess lipid accumulation in hepatocytes impairs their ability to respond to glucagon, with decreased amino acid catabolism and a compensatory hyperglucagonaemia (Wewer Albrechtsen *et al.* 2019). The multiplicative product of fasting glucagon and fasting alanine (glucagon–alanine index) increases with increasing hepatic insulin resistance and correlates with increasing liver transaminases (Wewer Albrechtsen *et al.* 2018*b*, Pedersen *et al.* 2020, Gar *et al.* 2021).

Hyperglucagonaemia is also observed in patients with insulin resistance (Wewer Albrechtsen *et al.* 2018*b*) and type 2 diabetes (Unger 1978). This is unsurprising because of the high degree of epidemiological and pathophysiological overlap between obesity, type 2 diabetes and MASLD, all of which are considered ‘metabolic-syndrome’-associated diseases (Huang 2009). For example, 87% of people diagnosed with type 2 diabetes are overweight or obese (Diabetes UK 2022) and 70% of subjects with obesity have MASLD (Williams *et al.* 2011). Where people with MASLD and type 2 diabetes are compared with weight-matched control people with type 2 diabetes but without MASLD, the presence of MASLD is associated with higher glucagon and amino acid levels (Junker *et al.* 2016, Wewer Albrechtsen *et al.* 2018*a*). This indicates that the driving force for hyperglucagonaemia and hyperaminoacidaemia is liver disease, which potentially could then contribute to the pathogenesis of diabetes via stimulation of hepatic glucose production (Unger 1978).

As well as direct effects of excess lipid potentially impairing hepatocyte sensitivity to glucagon, there are several additional potential causes for fasting and post-prandial hyperglucagonaemia in people with type 2 diabetes (Caruso *et al.* 2023). In this cohort, carbohydrate ingestion fails to suppress glucagon secretion but protein ingestion continues to stimulate glucagon secretion, even in the presence of hyperglycaemia (Müller *et al.* 1970). Insulin resistance of alpha cells may decrease the inhibitory effects of insulin on glucagon secretion; additionally, loss of beta-cell mass could decrease local levels of insulin in the fed state (Caruso *et al.* 2023).

Patients with cirrhosis of all causes also exhibit hyperglucagonaemia (Lewis *et al.* 1991, Bugianesi *et al.* 1998). Glucagon levels are higher in patients with cirrhosis than in patients with simple steatosis (Grandt *et al.* 2023) and worse in patients with cirrhosis and ascites than in those with cirrhosis without ascites (Lewis *et al.* 1991). Again, this could suggest that liver damage is a driver for hyperglucagonaemia. Indeed, plasma glucagon concentration correlates with liver function, as measured by elimination rate of antipyrine and caffeine (Lewis *et al.* 1991). In healthy volunteers, the transient glycaemia observed following glucagon injection is due to increased glycogenolysis (van Kempen *et al.* 2005); this increase in glycogenolysis is attenuated in patients with cirrhosis, even when they are glycogen replete (Bugianesi *et al.* 1998). Ureagenesis and hepatic nitrogen clearance during glucagon infusion are also reduced in patients with cirrhosis when compared to healthy controls, even when they are nutritionally compensated (Fabbri *et al.* 1993, Bugianesi *et al.* 1998).

### Other metabolic features in subjects with MASLD consistent with reduced glucagon signalling

Patients with MASLD have reduced hepatic expression of genes governing ureagenesis, amino acid and lipid metabolism (De Chiara *et al.* 2018, Eriksen *et al.* 2019, Suppli *et al.* 2020). Fasting total GLP-1 is elevated in patients with obesity and MASLD (Galindo Muñoz *et al.* 2015, Stinson *et al.* 2021); levels are highly correlated with liver transaminases (Stinson *et al.* 2021). Subjects with MASLD also have increased FGF-21 (Grandt *et al.* 2023). Pancreatic hypertrophy due to fat infiltration is associated with MASLD (Rugivarodom *et al.* 2022), but it is unknown whether patients with MASLD specifically have pancreatic alpha-cell hyperplasia.

One major difference between the models of glucagon signalling deficiency and people with MASLD is that the former have improved glucose tolerance compared to controls, whereas people with MASLD have a high prevalence of pre-diabetes and type 2 diabetes (Cao *et al.* 2024). One explanation is that MASLD is closely associated with insulin resistance and the resulting failure of insulin to lower circulating blood glucose masks any lowering effect of glucagon signalling deficiency on glucose levels. An additional possibility is that patients with MASLD are resistant to the actions of glucagon on amino acid catabolism but not to its effects on hepatic glucose production.

### Glucagon-stimulated effects in subjects with obesity or MASLD

Only a few glucagon infusion studies have been conducted in patients with metabolic-associated disease, and very few compare the effects of glucagon on metabolism in healthy volunteers with its effects on patients with obesity and/or MASLD (Table 1).

As with healthy volunteers, infusion of glucagon in patients with overweight/obesity is associated with an increase in plasma insulin and glucose (Vega *et al.* 2021). In a study comparing glucose turnover in response to a glucagon infusion between lean volunteers and patients with obesity, endogenous glucose production and rate of glucose disappearance were higher at baseline in lean subjects (due to greater insulin sensitivity). When expressed as an incremental change from baseline, there was no difference in glucose turnover in response to glucagon between healthy volunteers and people with obesity (Suppli *et al.* 2020). Another study comparing patients with obesity and MASLD to weight-matched controls without MASLD similarly found no differences in glucose production (Heebøll *et al.* 2022).

High-dose glucagon infusion (12.5 ng/kg/min or 25 ng/kg/min, giving plasma glucagon levels of around 85 pmol/L) reduces circulating amino acids in patients with obesity and increases urinary nitrogen excretion (Vega *et al.* 2021). However, while a lower dose of 3 ng/kg/h (giving high physiological plasma levels of 30pmol/L) decreased plasma amino acids in lean volunteers, it was not associated with a reduction in plasma amino acids in patients with obesity, half of whom had co-existing MASLD (Suppli *et al.* 2020). This could indicate that patients with obesity and MASLD are resistant to the effects of glucagon on amino acid uptake and catabolism.

In patients with obesity undergoing pancreatic clamp, high-dose glucagon infusion suppressed hepatic VLDL–triglyceride secretion, with a corresponding reduction in plasma VLDL–triglyceride levels. Both of these effects were attenuated in patients with MASLD of comparable weight (Heebøll *et al.* 2022).

### Evidence that glucagon sensitivity increases when MASLD improves

Weight loss is robustly associated with both improvement in MASLD (Koutoukidis *et al.* 2019) and reduction in fasting glucagon levels (Silvestre *et al.* 2017). Concurrent reductions in liver fat and surrogate markers of glucagon resistance (fasting glucagon, alanine and glucagon–alanine index) are observed after dietary weight loss (Winther-Sørensen *et al.* 2020), pharmacologically induced weight loss (Svane *et al.* 2022) and bariatric surgery (Pedersen *et al.* 2020, Pedersen *et al.* 2021, McGlone *et al.* 2023).

Reduction in liver fat does not necessarily cause improvement in glucagon sensitivity, however, as there are several potential confounders. A *post hoc* analysis of data from two dietary studies in patients with type 2 diabetes reported that although isocaloric and hypocaloric diets both led to a similar improvement in hepatic steatosis, only the latter was associated with improvement in glucagon–alanine index (Kjeldsen *et al.* 2023*a*). The hypocaloric, but not the isocaloric, diet was associated with an improvement in hepatic insulin sensitivity as evaluated by HOMA-IR, as well as with superior weight loss (Kjeldsen *et al.* 2023*a*). These findings could indicate that improvement in hepatic steatosis is not the only driver for an improvement in glucagon sensitivity. In a study where mice with diet-induced obesity were treated with sleeve gastrectomy or calorie restriction so that they lost the same amount of weight, surrogate markers of glucagon resistance improved only in the mice treated with sleeve gastrectomy (McGlone *et al.* 2023). Hepatic expression of genes downstream of glucagon activity (e.g. fatty acid synthase) were downregulated following sleeve gastrectomy but upregulated flowing calorie restriction. Taken together, these studies suggest improvement of glucagon sensitivity may not be a simple function of either weight loss or improvement in MASLD. It must be noted, however, that studies in this area to date have reported surrogate markers of glucagon sensitivity rather than direct measures.

## Molecular basis for glucagon resistance

There are several possible mechanisms via which glucagon resistance in hepatocytes could be mediated, these include reduction in plasma membrane GCGR expression; reduction in downstream signal transduction following GCGR stimulation; and allosteric modulation of glucagon receptor signalling. In genetic mouse models of glucagon resistance, in which mTORC1 signalling is upregulated, mice exhibit chronic hyperglucagonaemia associated with hyperglycaemia, and reduced glucose excursion in response to a glucagon tolerance test. In these mice, hepatic expression of the glucagon receptor was reduced when compared to controls (Bozadjieva Kramer *et al.* 2021, Lubaczeuski *et al.* 2023). When the inducible genetic upregulation was reversed, glycaemia improved and expression of GCGR returned to normal (Lubaczeuski *et al.* 2023). Further evidence that glucagon sensitivity is related to expression of the glucagon receptor comes from experiments involving chronic endurance training in rodents; this is associated with an increase in glucagon sensitivity alongside an increase in hepatocyte plasma membrane GCGR density compared to sedentary controls (Légaré *et al.* 2001, Melançon *et al.* 2013).

The stimulated glucagon receptor couples to G proteins which activate adenylyl cyclase (AC) activity to produce cAMP. In rats, increasing age was associated with a decrease in glucagon-stimulated cAMP production, alongside a reduction in stimulatory G protein content in hepatocytes and a reduction in intrinsic AC activity (Podolin *et al.* 2001). These changes were offset by endurance training. Glucagon receptor activity is known to be allosterically modulated by other cell membrane constituents (McGlone *et al.* 2021, Krishna Kumar *et al.* 2023); cholesterol content of hepatocytes is inversely related to glucagon receptor sensitivity *in vitro* and in mice (McGlone *et al.* 2022). Interestingly, hepatic cholesterol level is correlated with severity of hepatic steatosis (Min *et al.* 2012). It remains to be seen if modulation of GCGR signalling by cholesterol or other cell membrane constituents could mediate glucagon resistance in patients with MASLD.

## Conclusion

Glucagon signalling deficiency syndromes, as exhibited by patients with Mahvash disease, GCGR^−/−^ mice and pharmacological blockade of GCGR, are notable for very high plasma levels of glucagon and amino acids. Subjects have decreased expression of hepatic amino acid and lipid catabolism enzymes, along with improved glucose tolerance and insulin availability. They are resistant to weight gain on a high fat diet and may have increased propensity to hepatosteatosis. Some of these effects occur indirectly, often via an increase in GLP-1. Conversely, acute glucagon infusion decreases plasma amino acids, increases glucose output and decreases plasma cholesterol.

Many, but not all, of the baseline features of glucagon signalling deficiency are observed in patients with MASLD. There is limited evidence that the effect of glucagon infusion on amino acid catabolism is attenuated in patients with MASLD when compared to healthy volunteers. Interestingly, expected effects of reduced glucagon signalling on glucose tolerance are not observed in patients with MASLD either at baseline or in response to glucagon infusion. This could be due to co-existing insulin resistance masking glucagon-conferred improvements in glucose homeostasis, but further research is warranted. In particular, research should focus on the direct effects of glucagon on hepatic glucose, amino acid and lipid metabolism. The contribution of other hormones including insulin, as well as nutritional status, must be taken into consideration when planning and interpreting these studies.

If patients with MASLD are glucagon resistant, it remains to be seen whether glucagon resistance, via a reduction in hepatic lipid catabolism enzymes, contributes to the development of MASLD; or whether MASLD, possibly via hepatocyte fat accumulation modulating GCGR function, causes glucagon resistance. It is possible that there is a positive feedback loop in which both these scenarios co-exist. Early results of clinical trials of co-agonists of glucagon and its related receptors (GLP-1R and glucose-dependent insulinotropic peptide receptor) as treatments for MASLD are promising (American Diabetes Association 2023, Boehringer Ingelheim 2024). Breaking the cycle of glucagon resistance, by increasing glucagon signalling, could be a therapeutic rationale for the treatment of MASLD.

## Declaration of interest

TM-MT and SRB declare that they are shareholders in and consultants for Zihipp Ltd., an Imperial College spin-out company that develops gut hormone analogues for the treatment of obesity and associated metabolic disorders.

## Funding

This article presents independent research. The Section of Endocrinology and Investigative Medicine, Imperial College, is funded by grants from the MRC, NIHR and is supported by the NIHR Biomedical Research Centre Funding Scheme and the NIHR/Imperial Clinical Research Facility. The views expressed are those of the authors and not necessarily those of the funders, the NHS, the NIHR or the Department of Health. ERM, a clinical lecturer, is presently supported by the NIHR and the Academy of Medical Sciences. SRB is funded by the NIHR Imperial BRC. TT is funded by the NIHR, the NIHR Imperial BRC and the JP Moulton Charitable Foundation.
